# Neuroprotective and Memory-Enhancing Effect of the Combined Extract of Purple Waxy Corn Cob and Pandan in Ovariectomized Rats

**DOI:** 10.1155/2017/5187102

**Published:** 2017-07-09

**Authors:** Woranan Kirisattayakul, Jintanaporn Wattanathorn, Sittichai Iamsaard, Jinatta Jittiwat, Bhalang Suriharn, Kamol Lertrat

**Affiliations:** ^1^Department of Physiology and Graduate School (Neuroscience Program), Faculty of Medicine, Khon Kaen University, Khon Kaen 40002, Thailand; ^2^Integrative Complementary Alternative Medicine Research and Development Center, Khon Kaen University, Khon Kaen 40002, Thailand; ^3^Department of Physiology, Faculty of Medicine, Khon Kaen University, Khon Kaen 40002, Thailand; ^4^Department of Anatomy, Faculty of Medicine, Khon Kaen University, Khon Kaen 40002, Thailand; ^5^Faculty of Medicine, Mahasarakham University, Maha Sarakham 44150, Thailand; ^6^Faculty of Agriculture, Khon Kaen University, Khon Kaen 40002, Thailand

## Abstract

The neuroprotectant and memory enhancer supplement for menopause is required due to the side effects of hormone replacement therapy. Since purple waxy corn cob and pandan leaves exert antioxidant and acetylcholinesterase inhibition (AChEI) effects, we hypothesized that the combined extract of both plants (PCP) might provide synergistic effect leading to the improved brain damage and memory impairment in experimental menopause. To test this hypothesis, female Wistar rats were ovariectomized bilaterally and orally given various doses of the functional drink at doses of 20, 40, and 80 mg/kg for 28 days. The animals were assessed nonspatial memory using object recognition test every 7 days throughout the study period. At the end of study, they were assessed with oxidative stress status, AChEI, neuron density, and ERK1/2 signal in the prefrontal cortex (PFC). Interestingly, all doses of PCP increased object recognition memory and neuron density but decreased oxidative stress status in PFC. Low dose of PCP also decreased AChE activity while medium dose of PCP increased phosphorylation of ERK1/2 in PFC. Therefore, the improved oxidative stress status and cholinergic function together with signal transduction via ERK in PFC might be responsible for the neuroprotective and memory-enhancing effects of PCP.

## 1. Introduction

To date, the number of menopausal women is continually growing. The World Health Organization (WHO) has estimated that the number of menopausal women worldwide will be 1200 million within 2030 [1]. It has been reported that cognitive decline is one of the important symptoms frequently observed especially in premature menopause [2]. Unfortunately, the current therapeutic strategy is still not in satisfaction level. The effect of hormone replacement therapy (HRT) on the cognitive function of menopausal women is controversial [3–7]. In addition, serious adverse effect such as breast cancer risk is reported in HRT [8, 9]. Therefore, the alternative strategy has gained much attention.

Among various alternative strategies, plant-based therapy is very much popular [10]. In the recent years, the use of plant-based food supplement is increased in Thailand [11]. It has been demonstrated that dietary approaches are regarded as the safe and effective preventive intervention against neurodegeneration [12]. A pile of evidence has revealed that consumption of the polyphenol-rich supplements can enhance memory impairment [13–15]. Recent findings have demonstrated that the purple corn (*Zea mays* Linn., purple color) cob, an agricultural waste, can be served as an important natural resource of polyphenol [16]. It also exhibits potent antioxidant activity and can improve oxidative stress-related disorders [16, 17]. In addition to purple corn cob, pandan (*Pandanus amaryllifolius*), a commonly used culinary plant in Southeast Asia, also possesses high phenolic compound content and exhibits antioxidant activity [18]. An effervescent powder containing pandan also improves oxidative stress-related damage of the pancreas [19]. Based on these pieces of information and synergistic effect according to traditional folklore concept, the protective effect against oxidative stress-related brain damage and functional disorders of the combined extract of purple corn cob and pandan leaves (PCP) in menopausal women has been considered in order to produce an additive value of both plants. Currently, no data concerning this issue are available until now. Therefore, we aimed to determine the neuroprotective and memory-enhancing effects of the combined extract of purple corn cob and pandan leaves in experimental menopause in ovariectomized rats.

## 2. Materials and Methods

### 2.1. Chemicals and Reagents

Thiobarbituric acid (TBA), sodium dodecyl sulfate (SDS), glacial acetic acid, N-butanol, pyridine, 1,3,3-tetraethoxypropane (TEP), cytochrome C, xanthine oxidase, xanthine, glutathione reductase, nicotinamide adenine dinucleotide phosphate (NADPH), hydrogen peroxide, superoxide dismutase, glutathione peroxidase, catalase, acetylthiocholine iodide (ATCI), acetylcholinesterase, 5,5′-dithiobis (2-nitrobenzoic acid) (DTNB), cresyl violet, sodium acetate, sodium carbonate, 2,4,6-tripyridyl-striazine (TPTZ), Folin-Ciocalteu reagent, gallic acid, ascorbic acid, Trizma hydrochloride, potassium chloride, 2,2-diphenyl-1-picrylhydrazyl (DPPH), tris-hydrochloride, and sodium carbonate were purchased from Sigma-Aldrich (St. Louis, MO, USA). Chemicals used in Western blot analysis were purchased from Bio-Rad Laboratories. Methanol and acetic acid (HPLC grade) were purchased from Fisher Scientific.

### 2.2. Plant Material Preparation and Extraction

The cobs of purple waxy corn *(Zea mays*, open-pollinated cultivar) harvested during November–December 2012 were identified and kindly provided by Professor Kamol Lertrat and Assistant Professor Bhalang Suriharn, Department of Plant Science and Agricultural Resources, Faculty of Agriculture, Khon Kaen University, Khon Kaen, Thailand. Pandan (*Pandanus amaryllifolius*) leaves were harvested at the same period as *Z. mays* from the Khon Kaen province. The plant identification was performed by Mister Winai Somprasong, an expert agricultural scientist in the Botany and Plant Herbarium research group, Plant Varieties Protection Division, Ministry of Agriculture and Cooperatives. The cobs of *Z. mays* and the leaves of *P. amaryllifolius* were cleaned and cut into a small pieces; then, they were force dried by using an oven at 60°C overnight. The dried plants (2 kilograms of each plant) were twice extracted with 5 liters of distilled water. The percent yield of *Z. mays* and *P. amaryllifolius* were 2.4 and 8, respectively.

### 2.3. Preparation of a Polyphenol-Rich Functional Drink

Powder of various ingredients including 2% (*w*/*v*) combined extract of *Z. mays* and *P. amaryllifolius* (a ratio of both extracts was obtained from our unpublished in vitro data which provided optimum potential and under petit patent), 0.75% (*v*/*v*) sucralose, 1% (*v*/*v*) lemonade, 0.025% (*w*/*v*) salt, and 96.225% (*v*/*v*) water. All ingredients were mixed together and filtered through a cheesecloth, and the filtrate was used for further study.

### 2.4. Determination of Total Phenolic Compound Contents

The determination of total phenolic compounds content was carried out by using the Folin-Ciocalteu method [20]. In brief, an aliquot of combined extract beverage (20 *μ*l) was added to distilled water (1.58 ml) and 50% (*v*/*v*) Folin-Ciocalteu phenol reagent (0.1 ml) (Sigma-Aldrich). After 8 minutes of incubation, 20% sodium carbonate (0.3 ml) was added and mixed well. The mixture was kept in a dark room and incubated for 2 hours at room temperature. The absorbance was measured at 765 nm with a UV-spectrophotometer (Pharmacia LKB-Biochrom4060). Gallic acid at concentrations of 50–600 mg/l were used for preparing the standard calibration curve. The total phenolic compound was expressed as gallic acid equivalents per mg extract (mg/l GAE).

### 2.5. Assessment of DPPH Inhibition

The scavenging activity against free radicals of the developed drink was assessed via DPPH assessment. Briefly, 0.15 mM DPPH in methanol (0.5 ml) and the functional drink (1 ml) were mixed and incubated at room temperature for 30 minutes. The absorbance was determined at 517 nm with a UV-spectrophotometer (Pharmacia LKB-Biochrom4060). The DPPH radical scavenging activity was calculated using the following equation:

(1) $$\begin{array}{c} \%Inhibition\;of\;DPPH=\left[\left(\frac{Abs\;control-Abs\;sample}{Abs\;control}\right)\right]\times 100. \end{array}$$

Abs control was the absorbance of methanol plus DPPH reagent while Abs sample was the absorbance of developed drink or standard. The linear portion of percentage inhibition of combined extract beverage was plotted against its concentration. The half maximal inhibitory concentration (IC_50_) was calculated by using the equation from its graph [21]. All determinations were performed in triplicate.

### 2.6. Determination of Ferric-Reducing Antioxidant Power (FRAP) Assay

The assessment of ferric-reducing antioxidant power (FRAP) was performed based on the ability of the tested substance to reduce ferric tripyridyl triazine (Fe III TPTZ) complex to ferrous form (intense blue color) at low pH by using a modified method of Benzie and Strin [22]. FRAP reagent was freshly prepared by mixing solution A (300 mM acetate buffer pH 3.6), solution B (10 mM 2,4,6-tripyridyl-striazine (TPTZ) in 40 mM HCl), and solution C (20 mM ferric chloride) together at a ratio of A : B : C; 10 : 1 : 1, respectively, and kept in water bath at 37°C. The tested substance (50 *μ*l) was added to FRAP reagent (1.45 ml), mixed thoroughly, and incubated at 37°C for 10 minutes. The absorbance was measured with spectrophotometer at 593 nm (Pharmacia LKB-Biochrom4060). FRAP reagent and L-ascorbic acid (100–1000 *μ*M) were used as blank and standard calibration, respectively. Data were expressed as *μ*M L-ascorbic acid equivalent.

### 2.7. Determination of Anthocyanin Content

Anthocyanin content was determined according to the official method of the Association of Official Analytical Chemists (AOAC) [23]. The tested sample (1 ml) was mixed with 0.025 M potassium chloride pH 1.0 (2 ml) or 0.4 M sodium acetate pH 4.5 (2 ml). After the incubation at room temperature for 10 minutes, the absorbance was determined at 520 and 720 nm using a UV-spectrophotometer (Pharmacia LKB-Biochrom 4060). All assessments were performed as triplicate. Anthocyanin content was calculated and expressed as mg/l cyaniding-3-glucoside equivalent/mg extract (mg/l CGE) as follows:

(2) $$\begin{array}{c} Anthocyanin\;content\;\left(\frac{cyanindin-3-glucoside\;equivalent,mg}{l}\right)=\frac{\left(A\times MW\times DF\times 10^{3}\right)}{\left(ɛ\times 1\right)}, \end{array}$$

where A = (A 520 nm–A 700 nm) pH 1.0 − (A 520 nm–A 700 nm) pH 4.5, MW (molecular weight) = 449.2 g/mol, DF = dilution factor obtained from the study, *ɛ* = 26,900 molar extinction coefficient, in l mol^−1^ cm^−1^, for cyanindin-3-glucoside, 10^3^ = factor for conversion from g to mg, and l = path length of the cuvette in cm (1 cm).

### 2.8. Assessment of Acetylcholinesterase Inhibitory (AChEI) Activity

Inhibition of acetylcholinesterase of the sample was determined according to the method previously described [24] using acetylthiocholine chloride iodide (ATCI) as a substrate. In brief, combined extract beverage (25 *μ*l) was incubated with 15 mM ATCI (25 *μ*l), 3 mM DTNB (5,5′-dithiobis[2-nitrobenzoic acid]) (75 *μ*l), and 50 mM Tris buffer (pH 8) (50 *μ*l) for 5 minutes at room temperature. The absorbance was measured with a microplate reader (iMark™ Microplate Absorbance Reader) at 415 nm before and after adding 0.25 units/ml acetylcholinesterase (AChE) (25 *μ*l) to the mixture. The elevation of yellow color from the reaction was obtained and the percentage inhibition was calculated by comparing the yellow color of extract to a noninhibition well (Tris buffer). All tests were conducted in triplicate.

### 2.9. Fingerprint Chromatogram Assessment

The fingerprint chromatogram of the developed drink was analyzed by using gradient high-performance liquid chromatography (HPLC) system. High-performance liquid chromatography (HPLC) system consisted of 515 HPLC pump and 2998 photodiode array detector (Water Company, USA). Chromatographic separation was performed using Purospher®STAR, C-18 endcapped (5 *μ*m), LiChroCART®250-4.6, and HPLC-Cartridge, sorbet lot number HX255346 (Merk, Germany) with guard column (Merk, Germany). Methanol (A) and 7.5% acetic acid in deionized (DI) water (B) were used as mobile phases. The gradient elution was carried out at a flow rate of 1.0 ml/min with the following gradient: 0–17 min, 70%A; 18–22 min, 100%A; 23–25, 50%A; and 26–30 min, 60%A. The sample was filtered (0.45 *μ*m, Millipore), and a direct injection of tested sample at the volume of 20 *μ*l on the column was performed. The chromatograms were recorded at 280 nm using a UV detector and data analysis was performed using EmpowerTM3.

### 2.10. Experimental Animals and Protocols

Female Wistar rats (Laboratory Animal Center, Salaya, Nakhon Pathom, Thailand), weighing 200–250 g, were used as the experimental animals. They were randomly housed 6 per cage in a temperature-controlled room on a 12 h light/dark cycle with ad libitum access to food and water. All procedures in this experiment were strictly performed in accordance with the internationally accepted principles for laboratory use and care of the European Community (EEC directive of 1986; 86/609/EEC). The experiment protocols were approved by the Institutional Animal Care and Unit Committee, Khon Kaen University, Thailand (record number AEKKU 1/2556).

The experimental rats were divided into 8 groups (*n* = 6/group) as follows:

Group I: Naïve intact group; all rats received no treatment and were served as the control group.

Group II: Sham operation + vehicle; all rats were subjected to sham operation surgery and received distilled water which was served as the vehicle in this study.

Group III: OVX + vehicle; the experimental animals in this group were subjected to bilateral ovariectomy (OVX) and received the vehicle.

Group IV: OVX + donepezil (3 mg/kg BW); the OVX rats were orally given donepezil, an acetylcholinesterase inhibitor, at a dose of 3 mg/kg and were served as the positive control-treated group.

Group V: OVX + isoflavone; all OVX rats were orally given isoflavone, a well-known polyphenol substance with cognitive enhancing, at a dose of 20 mg/kg BW.

Groups VI–VIII: OVX + PCP20, OVX + PCP40, and OVX + PCP80; the OVX rats in these groups received PCP at doses of 20, 40, and 80 mg/kg BW, respectively.

The treatment programs of the assigned substances for rats in groups II–VIII were started since the first day after surgery and were maintained throughout a 28-day experimental period. All treatments were performed once daily in the morning with the total volume of 1.5 ml. The assessment of nonspatial memory was performed every 7 days throughout the study period while the determinations of oxidative stress markers including malondialdehyde (MDA), superoxide dismutase (SOD), catalase (CAT), glutathione peroxidase (GSH-Px), the activity of acetylcholinesterase (AChE), histology, and ERK1/2 expression in the prefrontal cortex were determined at the end of the study.

### 2.11. Ovariectomized Surgery Procedure

The experimental animal was anesthetized with sodium thiopental at the dose of 60 mg/kg BW via intraperitoneal route prior to the ovariectomy. The ovariectomized (OVX) procedure was performed according to the method which had been previously described [25]. Briefly, the dorsolateral incisions were performed bilaterally, the ovarian blood vessels were tied off, and the ovaries were removed. Then, the skin was sutured and the rat was returned to their cage after postoperation care. Sham operation was carried out with the same procedures except that both the ovaries were kept intact.

### 2.12. Object Recognition Test

The object recognition test, the common test for evaluating nonspatial memory in rats, was used to assess the effect of PCP on nonspatial memory. This test was performed as previously described elsewhere with minor modification [26]. In brief, each rat was placed in an open field (80 cm long × 50 cm high × 60 cm wide) with two identical objects for 3 minutes (T1) and then was placed back to its home cage. Both objects should be placed in a symmetric position in the central line of the area. Then, the animal was orally given the assigned substance, and 30 minutes later, the second 3-minute trial was performed. In this session, one of the objects was replaced with the novel object which was totally different in shape and size at the same location. During the intertrial interval, the objects and open-field apparatus object were cleaned with 70% ethanol to avoid a confounding error induced by the influence of odor. The amount of time which the rat spent exploring each object was recorded and calculated as a novel object ratio (NOR) as the following equation:

(3) $$\begin{array}{c} NOR=\frac{\left(T_{novel}-T_{familiar}\right)}{\left(T_{novel}+T_{familiar}\right)}, \end{array}$$

where *T*_novel_ = time spent to explore the novel object and *T*_familiar_ = time spent to explore the familiar object.

### 2.13. Histological Study

After perfusion, the brains were removed and fixed with 4% paraformaldehyde solution (pH 7.4) at 4°C. Then, they were cryoprotected in formalin-sucrose (30%) for 2-3 days. Serial sections of tissue containing prefrontal cortex were cut frozen on a cryostat (Thermo Scientific™ HM 525 Cryostat) at 20 *μ*m thick and mounted on slides coated with 0.3% aqueous solution of gelatin containing 0.05% aluminum potassium sulfate. To stain with cresyl violet, the slides were air dried; hydrated by successive immersion in 95, 70, and 50% ethanol; stained in 0.5% cresyl violet for 2 min at room temperature; dehydrated by successive immersion in 50, 70, 95, and 100% ethanol and xylene; and mounted with DPX. Three representative slides containing the prefrontal cortex were selected according to the stereotaxic coordinates anteroposterior 2.5–4.5 mm and mediolateral 0.2–1.0 mm from the rat brain atlas [27]. The analysis was performed by a blinded observer. The evaluation was performed via Olympus light microscope model BH-2 (Japan) at 40x magnification. The density of survival neurons in medial prefrontal cortex area (mPFC) was expressed as number of cells/255 *μ*m^2^.

### 2.14. Determination of Extracellular Signal-Regulated Kinase 1/2 (ERK1/2) Expression

The prefrontal cortex was subjected to a 2-minute homogenization in 1/10 (*w*/*v*) M-PER mammalian protein extraction (Pierce Protein Biology Product, Rockford, IL, USA) containing protease inhibitor cocktail (Sigma-Aldrich). Then, it was centrifuged at 14,000*g* for 10 minutes at 4°C. The supernatant was harvested and used for the determination of ERK1/2 expression. Protein concentration of the supernatant was quantified by using NanoDrop instrument (Thermo Fisher Scientific, Wilmington, Delaware USA). Total 30 *μ*g of brain samples were separated by 10% sodium dodecyl sulfate polyacrylamide gel electrophoresis (SDS-PAGE) at 80 V. All protein bands were transferred.

The determination of ERK1/2 protein was performed according to the method previously described with a minor modification [28]. Total of 30 *μ*g of brain samples were separated by 10% sodium dodecyl sulfate polyacrylamide gel electrophoresis (SDS-PAGE) at 80 V. Proteins from the gel were transferred to a nitrocellulose membrane (Bio-Rad Laboratories) and blocked with 5% nonfat dry milk in 0.1% tween 20 tris-buffered saline (TBS-T) for 30 minutes. After the blocking of a membrane, they were incubated with primary antibody which recognized ERK1/2 or monoclonal rabbit antiphosphorylated p44/p42 mitogen-activated protein kinase (MAPK) (Cell Signaling Technology Inc.; dilution, 1 : 1000) for 2 hours at room temperature. Then, they washed and incubated with secondary antibody conjugated with horseradish peroxidase or anti-rabbit IgG, HRP-linked antibody (Cell Signaling Technology Inc.; dilution, 1 : 2000) for 2 hours at room temperature. The band density was detected with an enhanced chemiluminescent (ECL) system (GE Healthcare, Piscataway, NJ). The analysis was performed using ImageQuant TL analysis software (GE Healthcare, Piscataway, NJ). The expression was normalized using antitotal ERK1/2. Data were presented as a relative density to the naïve control.

### 2.15. Biochemical Assays

The prefrontal cortex, an important area in learning and memory, was isolated and prepared as brain homogenate by subjecting to homogenization with 50 volume of 0.1 M phosphate buffer saline. Then, the homogenate was used for the determination of acetylcholinesterase (AChE) activity and oxidative stress status including malondialdehyde (MDA) level and the activities of superoxide dismutase (SOD), catalase (CAT), and glutathione peroxidase (GSH-Px). Protein concentration was assessed according to the Lowry method [29] and albumin bovine serum (2–20 mg/ml) was used as a standard.

The determination of AChE was carried out to reflect the cholinergic function in OVX rats by the colorimetric method [30]. A reaction mixture of 200 *μ*l of 0.1 mM sodium phosphate buffer (pH 8.0), 10 *μ*l of 0.2 M DTNB (5,5′-dithio-bis-(2-nitrobenzoic acid)), and 20 *μ*l of the sample solution were incubated for 5 minutes, and the absorbance at 415 nm was recorded via microplate reader (iMark Microplate Absorbance Reader). Then, 10 *μ*l of acetylcholine thiochloride (ACTI) was added, incubated for 3 minutes, and recorded the absorbance at 415 nm. The activity of AChE was calculated according to the equation below and expressed as mmol/min/g protein.

(4) $$\begin{array}{c} AChE\;activity=\left(∆A/1.36\times 10^{4}\right)\times 1/\left(20/230\right)C, \end{array}$$

where ∆*A* = the difference of absorbance/minute and *C* = protein concentration of brain homogenate.

MDA level was assessed according to the method of Thiraphatthanavong et al. [31]. In brief, the mixture of 0.1 ml of brain homogenate, 0.1 ml of 8.1% (*w*/*v*) sodium dodecyl sulfate, 0.75 ml of 20% (*v*/*v*) acetic acid pH 3.5, 0.75 ml of 0.8% (*w*/*v*) thiobarbituric acid, and 0.3 ml of distilled water were mixed thoroughly and boiled at 95°C for 1 hour. After cooling, 0.5 ml of water and 2.5 ml of the mixture of n-butanol and pyridine at the ratio of 15 : 1 were added, mixed together, and centrifuged at 4000 rpm for 10 minutes. The pink layer was harvested and determined the optical density at 532 nm. 1,1,3,3-tetramethoxypropane (2–20 nmol) was served as a standard and MDA level was expressed as nmol/mg protein.

SOD assessment was performed according to the method previously described elsewhere [32]. Briefly, 20 *μ*l of brain homogenate was mixed with the mixture which contained 216 mM potassium phosphate buffer (KH_2_PO_4_), 10.7 mM ethylenediaminetetraacetic acid, 1.1 mM cytochrome C, and 0.54 mM xanthine solution pH 7.4 at the ratio of 25 : 1 : 1 : 50. Then, 20 *μ*l of 0.05 units/ml of xanthine oxidase was added and incubated for 5 minutes at room temperature. The absorbance was measured at 490 nm using microplate reader. SOD enzyme activities at the concentrations of 0–10 units/ml were used as standards, and the results were expressed as units/mg protein.

The activity of catalase (CAT) was evaluated indirectly by measuring the residual H_2_O_2_ which was titrated by potassium permanganate. In brief, 10 *μ*l of brain homogenate was mixed with 50 *μ*l of 30 mM H_2_O_2_, 25 *μ*l of 5 N H_2_SO_4_, and 150 *μ*l of 5 mM KMnO_4_. The mixture was shaken and the absorbance was measured at 490 nm. CAT enzyme at the concentration range of 0–10 units/ml was used as a standard and the result was expressed as units/mg protein [32].

Glutathione peroxidase activity was assessed using the colorimetric method. In brief, 10 *μ*l of brain homogenate was mixed with the mixture containing 50 *μ*l of 30 mM H_2_O_2_, 25 *μ*l of 5 N H_2_SO_4_, and 150 *μ*l of 5 mM KMnO_4_. The mixture was shaken and the absorbance was measured at 490 nm. The standard calibration curve was prepared by using CAT enzyme at the concentration range of 0–10 units/ml. CAT activity was expressed as units/mg protein [32].

### 2.16. Statistical Analysis

Data are presented as mean ± standard error of mean (SEM). The statistical analysis of the experiment was carried out using IBM SPSS Statistics (version 21). Data was analyzed using one-way analysis of variance (ANOVA), followed by Tukey's post hoc test. Probability levels less than 0.05 were accepted as significant.

## 3. Results

### 3.1. Biological Activities of the Combined Extract

Total phenolic compounds and anthocyanin content together with the biological activities including the antioxidant activity (DPPH and FRAP assay) and acetylcholinesterase inhibition activity of the combined extract were evaluated. The results showed that 1 ml of the combined extract contained the total phenolic compounds and anthocyanin contents of 184.00 ± 1.91 mg/l gallic acid equivalent and 25.66 ± 0.32 mg/l cyanidin-3-glucoside equivalent, respectively. IC_50_ of the antioxidant activity via 2,2-diphenyl-1-picrylhydrazyl (DPPH) assay was 56.37 ± 0.45 *μ*g/ml, whereas the antioxidant activity via ferric-reducing antioxidant power (FRAP) assay was 602.40 ± 2.33 *μ*M L-ascorbic acid equivalent. In addition, AChEI activity showed IC_50_ at a concentration of 1950 ± 16.02 *μ*g/ml as shown in Table 1.

### 3.2. The Fingerprint of the Combined Extract

**Figure 1:**
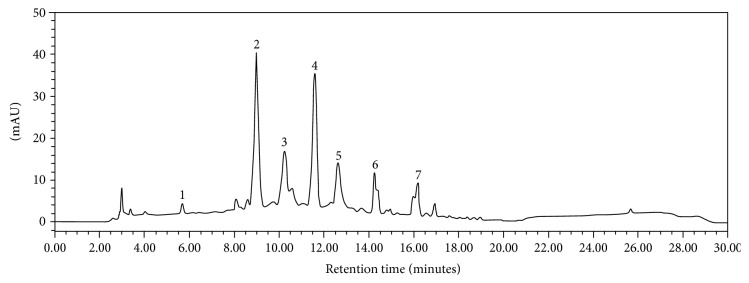
The fingerprint chromatogram of the PCP using HPLC analysis.

Figure 1 shows the fingerprint chromatogram of PCP, the combined extract of purple corn cob and pandan leaves. More than 7 different peaks were observed in the chromatogram. Four of them were anthocyanins (peak 2–peak 5) including cyaniding-3-glucoside (peak 2), pelargonidin-3-glucoside (peak 3), cyanidin 3-O-(6″-malonyl-glucoside) (peak 4), and cyaniding-3-o-B-glucopyranoside (peak 5). It was found that the contents of anthocyanins mentioned earlier in PCP were 3.239 ± 0.014, 2.543 ± 0.011, 2.993 ± 0.024, and 2.335 ± 0.006 mg/ml, respectively. In addition to anthocyanins, gallic acid, rutin, and ferulic acid were observed at the concentrations of 0.180 ± 0.001, 0.337 ± 0.001, and 0.341 ± 0.027 mg/ml, respectively.

### 3.3. Effect of the Combined Extract on Nonspatial Memory in OVX Rats

**Figure 2:**
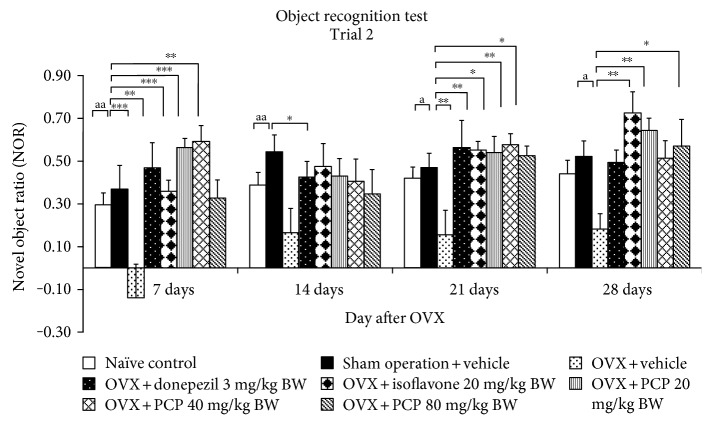
The effect of the PCP on nonspatial memory assessing by the object recognition test (*n* = 6/group). Data were expressed as mean ± SEM. ^a, aa^*p* value < 0.05, 0.01, respectively, compared with the sham operation group. ^∗^, ^∗∗^,  ^∗∗∗^*p* value < 0.05, 0.01, and 0.001, respectively, compared with the OVX + vehicle-treated group.

Memory-enhancing effect of the combined extract on nonspatial memory was shown in Figure 2. It was found that sham operation rats showed no significant change on novel object ratio (NOR). OVX rats treated with vehicle significantly decreased NOR throughout the study period (*p* value < 0.01, 0.01, 0.05, and 0.05, resp., compared with the sham operation group). OVX rats with isoflavone treatment attenuated the reduction of NOR induced by OVX throughout the study period (*p* value < 0.01, 0.05, 0.01, and 0.01, resp., compared with OVX + vehicle). Donepezil also showed the mitigation effect on the reduction of NOR in OVX rats, but significant differences were observed only at 7- and 21-day treatment periods (*p* value < 0.001 and 0.01, resp., compared with the OVX + vehicle-treated group). The combined extract at doses of 20 and 80 mg/kg BW significantly attenuated the decreased NOR in OVX rats at 7, 21, and 28 days of treatment (*p* value < 0.001 and 0.01; 0.05 all; and 0.01 and 0.05, resp., compared with the OVX + vehicle-treated group). The significant mitigation effect of PCP at a dose of 40 mg/kg BW on NOR was also observed at 7 and 21 days of treatment (*p* value < 0.001 and 0.01, resp., compared with the OVX + vehicle-treated group). The increased NOR in the PCP treatment groups were also observed at a 14-day study period, but no significant difference was revealed.

### 3.4. Histological Change in the Prefrontal Cortex

**Figure 3:**
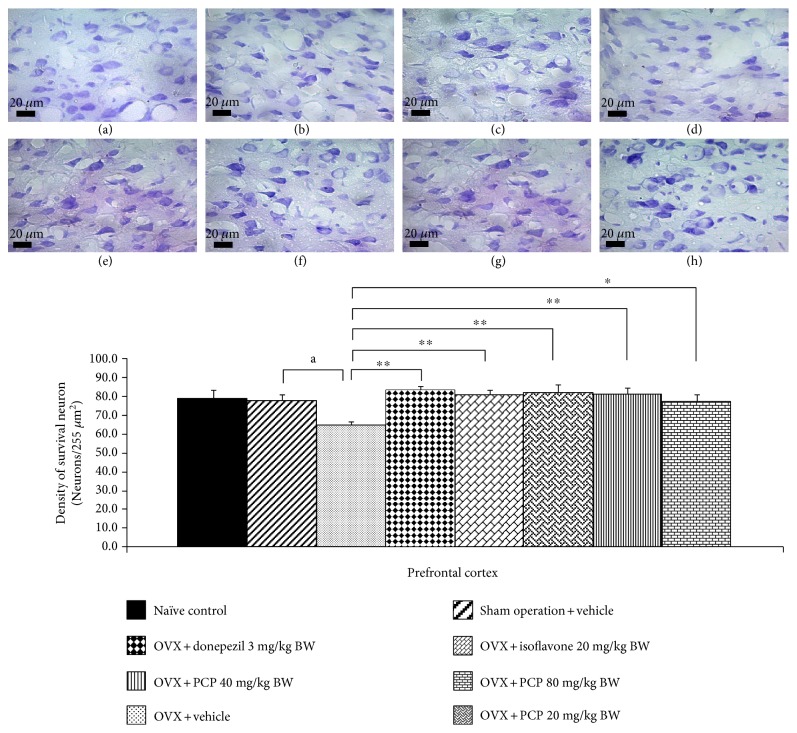
The effect of the PCP on the density of survival neurons in medial prefrontal cortex (mPFC) (*n* = 6/group). The upper panel showed the photomicrograph of the coronal section of rat brains in (a) naïve control, (b) sham operation + vehicle, (c) OVX + vehicle, (d) OVX + donepezil, (e) OVX + isoflavone, (f) OVX + PCP 20 mg/kg BW, (g) OVX + PCP 40 mg/kg BW, and (h) OVX + PCP 80 mg/kg BW. Scale bar: 20 *μ*m. The lower panel demonstrated the density of survival neurons of mPFC. ^a^*p* value < 0.05, compared with the sham operation group. ^∗^,  ^∗∗^*p* value < 0.05 and 0.01, respectively, compared with the OVX + vehicle-treated group. Data were expressed as mean ± SEM.

Based on the previous finding that the prefrontal cortex played a crucial role on working memory in rodents [33], we also investigated the neuron density in this area and results were shown in Figure 3. The current results demonstrated that OVX treated with vehicle significantly reduced the neuron density in PFC (*p* value < 0.05, compared with the sham operation group). Both donepezil and isoflavone could attenuate the reduction of neuron density in PFC of OVX rats (*p* value < 0.01 all; compared with the OVX + vehicle-treated group). In addition, combined extract at the dosage range used in this study also significantly attenuated the reduction of neuron density in PFC of OVX rats (*p* value < 0.01, 0.01, and 0.05; compared with the OVX + vehicle-treated group).

### 3.5. Biochemical Assays

Table 2 shows the effect of PCP on oxidative stress markers including MDA level and the activities of SOD, CAT, and GSH-Px. Sham operation showed no significant changes of the mentioned parameters. OVX rats which received vehicle significantly increased MDA level (*p* value < 0.05; compared to the sham operation group) in the prefrontal cortex (PFC). Donepezil treatment failed to produce the significant changes on all parameters just mentioned in OVX rats. However, isoflavone significantly decreased MDA level but enhanced SOD activity (*p* value < 0.05 and 0.01, resp., compared to the OVX + vehicle-treated group). Interestingly, PCP at all doses used in this study significantly decreased MDA level in PFC (*p* value < 0.001 all; compared to the OVX + vehicle-treated group). The elevation of SOD activity in PFC was observed in OVX rats which received PCP at the concentrations of 20 and 40 mg/kg BW (*p* value < 0.001 and 0.01, resp., compared with the OVX + vehicle-treated group).

**Figure 4:**
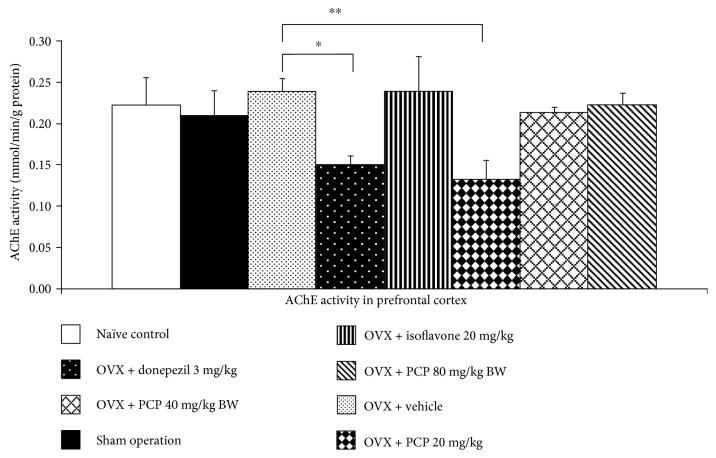
The effect of PCP on the activity of AChE in the prefrontal cortex (*n* = 6/group). ^∗^,  ^∗∗^*p* value < 0.05 and 0.01, respectively, compared with the OVX + vehicle-treated group. Data were expressed as mean ± SEM.

Since the cholinergic system plays a crucial role on learning and memory [34], we also investigated the effect of PCP on the cholinergic system by using the suppression activity of AChE or AChEI activity as an indirect index, and results were shown in Figure 4. It was found that both sham operation and OVX rats which received vehicle failed to produce the significant changes of AChE. The decreased AChE activity was observed in OVX rats which received donepezil and combined extract at the dose of 20 mg/kg BW (*p* value < 0.05 and 0.01, resp., compared with the OVX rats + vehicle-treated group). When compared with OVX rats which received vehicle, no significant changes of AChE were observed in the other groups.

### 3.6. ERK1/2 Signaling Pathway

**Figure 5:**
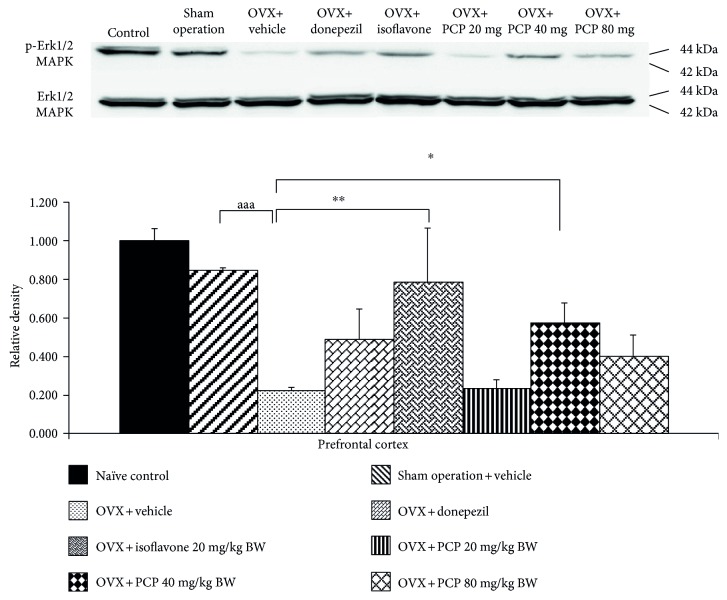
The effect of the PCP on the expression of phosphorylation ERK1/2 (p-ERK1/2) protein in the prefrontal cortex (*n* = 6/group). ^aaa^*p* value < 0.001, compared with the sham operation group. ^∗^,  ^∗∗^*p* value < 0.05 and 0.01, respectively, compared with the OVX + vehicle-treated group. Data were expressed as mean ± SEM.

Based on the crucial role of ERK1/2 on the survival neurons and memory enhancement [28], the effect of PCP on the phosphorylation ERK1/2 was also determined, and results were shown in Figure 5. The current data showed that OVX rats significantly decreased expression of phosphorylation ERK1/2 in PFC (*p* value < 0.001; compared with the sham operation group). Isoflavone and medium dose of PCP produced the significant attenuation effect induced by OVX (*p* value < 0.01 and 0.05, resp., compared with the OVX + vehicle-treated group).

## 4. Discussion

The current data showed that ovariectomy, a widely used model of menopause, increased oxidative stress status, impairment of the cholinergic system, and memory impairment which were in concordance with the previous study [35, 36]. In the present study, it was found that OVX increased MDA without changes of the main scavenger enzymes such as SOD, CAT, and GSH-Px. This was in agreement with the previous study [37]. These results suggested that the elevation of MDA level might occur either via the increased oxidative stress production or via the decreased function of the nonenzymatic antioxidant system. Interestingly, PCP, the combined extract of purple corn cob and pandan leaves attenuated the memory impairment evaluated by using the object recognition test.

It has been reported that the prefrontal cortex plays a crucial role on nonspatial memory. Lesion of this area could induce nonspatial memory impairment [38]. In this study, we have found that this impairment was attenuated by both isoflavone and all doses of PCP. The reduction of MDA level which indicated the decreased oxidative stress status and the enhanced neuron density was also observed in OVX rats which received isoflavone and all doses of PCP. Therefore, we suggested that PCP and isoflavone might improve oxidative stress status leading to the enhanced neuron density in PFC resulting in the improved nonspatial memory. The decreased oxidative stress status in PFC observed in this study might occur partly via the enhanced function of a scavenger enzyme especially SOD. Since no closed relationship between the decreased MDA level and the enhanced SOD activity in PFC was observed, other factors such as the decreased nonenzymatic system and the decreased oxidative stress production mentioned earlier might also contribute to the role.

In addition to oxidative stress, the cholinergic system in PFC also plays the crucial role on memory. Depletion of acetylcholine (ACh) together with the elevation of acetylcholinesterase (AChE) in the PFC induces memory impairment both in primates and in rodents [39, 40]. The cognitive-enhancing effect of donepezil, an AChEI, was observed without changes of oxidative stress markers. Our study also demonstrated that both donepezil and low dose of PCP also suppressed AChE activity in PFC. Therefore, the enhanced cholinergic function in PFC by suppressing AChE activity in the mentioned area also contributes to a role on the cognitive-enhancing effect of both substances. It has been reported that polyphenol [14] including anthocyanins [41] can improve memory impairment induced by scopolamine which exerts the effect at muscarinic receptors. Since PCP contains polyphenols and anthocyanins, it is also possible that PCP also exerts its influence on muscarinic receptor. However, this requires further investigation.

Recently, it has been demonstrated that mitogen-activated protein kinase (MAPK) especially ERK1/2 contributes to the pivotal role on learning and memory [42]. The substances which improve ERK signaling also improve the impairment of object recognition memory [43]. Based on this information, we did suggest that the cognitive-enhancing effect of isoflavone and medium dose of PCP might also occur via the enhanced ERK1/2 signaling pathway.

Anthocyanins, a member of flavonoids, have been shown to exert the neuroprotective and cognitive-enhancing effect [44]. Since our fingerprint of PCP showed that the main ingredient in PCP was anthocyanins (peak 2–peak 5), we suggested that the neuroprotective effect and cognitive-enhancing effects of PCP in this study might involve anthocyanins. However, the effect of other ingredients still cannot be omitted.

Our data failed to show a dose-dependent manner of PCP. The possible explanation might be due to the nonsimple linear relationship between the concentration of PCP and the observed parameters. Since many factors exert the influences on the observed parameters in this study, no simple linear relationship was observed. In addition, PCP contained many ingredients, so the effect of an active ingredient could be masked by other ingredients.

In this study, we have found that our data showed that although in vitro data showed that IC_50_ of AChEI of PCP was very high, low dose of PCP could exert the cognitive-enhancing effect via the suppression of AChE in the prefrontal cortex while the medium and high doses of PCP failed to exert this effect. The possible explanation might also occur as that mentioned earlier in the lack of a dose-dependent study.

Taken all data together, our study highlights the neuroprotective and cognitive effects of PCP that might occur primarily via the decreased oxidative stress which in turn increased neuron density in the brain especially in PFC, an area playing an important role on learning and memory especially nonspatial memory, resulting in the improved nonspatial memory. However, the improved cholinergic function and signal transduction via ERK1/2 might also exert the roles especially at low and medium doses, respectively.

## 5. Conclusion

This study is the first study to demonstrate the neuroprotective and cognitive effects of PCP. We have shown that the combined extract of purple corn cob and pandan leaves can be served as functional ingredients for developing neuroprotectant and cognitive enhancer for menopausal women. Therefore, we highlight how to create the value for agricultural waste such as purple corn cob. However, chronic toxicity is required in order to assure the consumption safety before moving forward to a clinical trial study.

## Figures and Tables

**Table 1:** The biological activity of PCP including total phenolic compound, anthocyanin content, DPPH radical activity, FRAP activity, and AChEI activity.

Test	PCP	Standard reference
Total phenolic compound (mg/l GAE)	184 ± 1.91	—
Anthocyanin content (mg/l CGE)	25.66 ± 0.32	—
FRAP activity (*μ*M L-ascorbic acid equivalent)	602.40 ± 2.33	—
DPPH radical activity (IC_50_*μ*g/ml)	56.37 ± 0.45	Ascorbic acid 2.89 ± 0.01
AChEI activity (IC_50_*μ*g/ml)	1950 ± 16.02	Donepezil 0.51 ± 0.03

**Table 2:** The effect of PCP on oxidative stress markers in the prefrontal cortex (*n* = 6/group).

	Oxidative stress markers in the prefrontal cortex			
Treatment	MDA	GSH-Px	SOD	CAT
	(nmol/mg protein)	(Units/mg protein)	(Units/mg protein)	(Units/mg protein)
Naïve control	0.073 ± 0.0.001	0.661 ± 0.034	0.567 ± 0.164	2.707 ± 0.234
Sham operation	0.077 ± 0.007	0.576 ± 0.052	0.834 ± 0.256	2.516 ± 0.237
OVX + vehicle	0.095 ± 0.008^a^	0.523 ± 0.034	0.309 ± 0.100	2.684 ± 0.216
OVX + donepezil 3 mg/kg BW	0.090 ± 0.007	0.714 ± 0.047	1.387 ± 0.344	2.650 ± 0.187
OVX + isoflavone 20 mg/kg BW	0.059 ± 0.005^∗^	0.902 ± 0.111	2.220 ± 0.458^∗∗^	2.130 ± 0.210
OVX + PCP 20 mg/kg BW	0.047 ± 0.012^∗∗∗^	0.866 ± 0.186	3.080 ± 0.352^∗∗∗^	2.447 ± 0.359
OVX + PCP 40 mg/kg BW	0.034 ± 0.002^∗∗∗^	0.799 ± 0.070	2.047 ± 0.184^∗∗^	2.151 ± 0.160
OVX + PCP 80 mg/kg BW	0.032 ± 0.004^∗∗∗^	0.639 ± 0.029	1.068 ± 0.366	1.921 ± 0.092

^a^ *p* value < 0.05, compared with the sham operation group. ^∗^, ^∗∗^,  ^∗∗∗^*p* value < 0.05, 0.01, and 0.001, respectively, compared with the OVX + vehicle-treated group. Data were expressed as mean ± SEM.
